# Beat-to-beat aortic valve opening detection from impedance cardiography using machine learning

**DOI:** 10.3389/fdgth.2026.1944092

**Published:** 2026-10-07

**Authors:** Luca Abel, Sebastian Stühler, Tobias Steigleder, Christoph Ostgathe, Nicolas Rohleder, Bjoern M. Eskofier, Robert Richer

**Affiliations:** 1 Department Artificial Intelligence in Biomedical Engineering (AIBE), Friedrich-Alexander-Universität Erlangen-Nürnberg (FAU), Erlangen, Germany; 2 Munich Center for Machine Learning (MCML), Munich, Germany; 3 Department for Palliative Medicine, University Hospital Erlangen, Erlangen, Germany; 4 Chair of Health Psychology, Department of Psychology, Friedrich-Alexander-Universität Erlangen-Nürnberg (FAU), Erlangen, Germany; 5 Chair of AI-Supported Therapy Decisions, Institute for Medical Information Processing, Biometry, and Epidemiology, LMU München, Munich, Germany; 6 Translational Digital Health Group, Institute of AI for Health, Helmholtz Zentrum München, Neuherberg, Germany

**Keywords:** aortic valve opening, impedance cardiogram (ICG), machine learning, physiology, pre-ejection period

## Abstract

**Introduction:**

Accurate detection of the aortic valve opening, represented by the B-point in the impedance cardiogram (ICG), is a critical step in non-invasive hemodynamic monitoring. The B-point is essential for deriving key parameters like the pre-ejection period (PEP) and left ventricular ejection time (LVET). However, accurately identifying this fiducial point is challenging due to a high intra-individual difference in the waveform, interfering factors like body movement, and a historical lack of publicly available datasets for evaluation.

**Methods:**

To overcome this research gap, we propose a novel machine learning-based approach for beat-to-beat B-point detection that leverages domain knowledge from 12 different established ICG-based fiducial point detection algorithms. Based on this feature set, we trained different machine learning pipelines on over 11,000 cardiac cycles from 39 young and healthy individuals included in our publicly available datasets.

**Results:**

The best-performing pipeline, based on a Random Forest Regressor, achieved a considerable improvement in B-point detection accuracy and robustness with a mean absolute error (MAE) of 8.13 ± 12.12 ms.

**Discussion:**

Compared to the best-performing traditional algorithm, our approach significantly reduced the MAE by 54.7% and its standard deviation by 47.4%. Furthermore, the achieved error is lower than the inter-rater error (8.35 ± 14.78 ms). Our novel B-point detection method is available for the research community through integration into our open-source framework PEPbench.

## 1 Introduction

Accurate measurement of hemodynamic parameters, such as the pre-ejection period (PEP), left ventricular ejection time (LVET), and stroke volume (SV), relies on the reliable detection of the aortic valve opening. This physiological event is marked by the B-point within the first derivative of the impedance cardiogram (ICG), conventionally denoted as dZ/dt (1, 2). The parameters, such as PEP and LVET, serve as crucial non-invasive indices of myocardial contractility and autonomic nervous system regulation in both clinical monitoring and psychophysiological research (3, 4).

Given that manual B-point detection is time-intensive and susceptible to inter-rater variability, making it impractical for clinical application (5), considerable effort has been put into the development of automatic B-point detection algorithms in recent years. However, the automatic detection of the B-point poses a significant challenge due to several factors. The morphology of the B-point can exhibit substantial inter- and intra-individual variation (3, 5–8). These variations can manifest as subtle inflections in the dZ/dt signal, which are inherently difficult to identify (1). Furthermore, the ICG waveform can be distorted by interfering factors, including respiration, body movement, and underlying cardiovascular dysfunction, thereby complicating reliable automated B-point detection (1, 3, 9).

A common approach for automatic B-point detection is the use of ensemble averaging, a technique that reduces noise and artifacts to simplify the reliable identification of fiducial points. However, this method has notable drawbacks: it can lead to the loss of relevant waveform details and is not suitable for real-time applications (10–13). While beat-to-beat B-point detection addresses these issues, it is considerably more challenging than B-point detection on ensemble-averaged signals. Nevertheless, a wide variety of algorithms were proposed in recent years to address this beat-to-beat detection problem.

The most prominent family of beat-to-beat extraction algorithms applies predefined rules on the dZ/dt signal or its first- ($d^{2}Z/dt^{2}$) and second- ($d^{3}Z/dt^{3}$) order derivatives to identify the point corresponding to a specific, or the most appropriate morphological pattern of the actual B-point (3, 5–8, 14–20). Other methods have employed weighted windows that transform the signal to emphasize specific sections of the waveform (21), adaptive search windows to constrain the search area (2), matched filters applied to signal templates (9), and empirical mode decomposition (22). Furthermore, a variety of algorithms based on time-frequency analysis (23) and wavelet transformations (9, 19, 24, 25) have been utilized. Similarly, data-driven B-point detection algorithms, using linear and quadratic regression formulas, have also been proposed to estimate the B-point location (1). Despite the variety of algorithms, so far, no algorithm has been established as the recommended one, unlike for R-peak detection in the ECG, where an algorithm originally proposed by Pan & Tompkins (26), which was continuously improved over the years, has been established as best practice. The primary reason for this is the lack of reproducibility, as the algorithm implementations and the data used for evaluation are often not publicly disclosed. This has made it difficult to conduct a fair comparison among the proposed algorithms. Moreover, the long-standing absence of a publicly available database with annotated fiducial points may explain why machine learning (ML) has not been widely adopted in this field of research, in contrast to its prevalence in other domains (27).

To the best of our knowledge, machine learning and deep learning algorithms for B-point detection have been explored in only three published studies so far. Cieslak et al. (12) reported a mean absolute error (MAE) of 1.3 ± 1.5 ms, the model proposed by Sheikh et al. (28) achieved a MAE of 3.5 ± 9.2 ms, and the deep learning model developed by Wang et al. (29) results in a MAE of around 5 ms. While these results are promising, all approaches have major limitations. The models were either trained on ensemble-averaged data (12, 28) or the datasets used for training and evaluation were relatively small (29) (417 cardiac cycles) or were not published alongside (28, 29).

A major obstacle in developing and comparing B-point detection methods, the lack of publicly available, annotated data, has recently been addressed. Several new datasets containing annotated dZ/dt signals are now accessible, including those from Miljković & Šekara and Pale et al. (8, 21). Furthermore, we published two datasets featuring both annotated electrocardiogram (ECG) and dZ/dt signals in previous work: EmpkinS (30) and Guardian (31).

Building on these publicly available datasets, we proposed the *PEPbench* framework for systematic benchmarking of B-point detection algorithms (27). Utilizing this, we systematically evaluated most of the existing B-point detection algorithms in the field. Our results showed that, on the EmpkinS dataset, the algorithm proposed by Drost et al. (18) performed best with a MAE of 14.9 ± 14.7 ms. In contrast, the linear regression-based algorithm developed by Lozano et al. (1) reported the lowest MAE on the Guardian dataset, 16.7 ± 14.8 ms. This comprehensive benchmarking was a major step forward for standardizing the comparison of B-point detection methods. Building on that, we set out to improve the B-point detection by combining the strengths of existing algorithms to achieve unprecedented accuracy for beat-to-beat B-point detection using machine learning.

In this paper, we introduce an ML-based approach for automated, beat-to-beat B-point detection, as outlined in overview Figure 1. We trained and evaluated our proposed model on the same two publicly available datasets (EmpkinS and Guardian) from our previous work, utilizing over 11,000 cardiac cycles from 39 individuals for a large-scale, direct comparison against already established algorithms (27). We leveraged the existing domain knowledge inherent in published B-point detection algorithms, each possessing unique strengths and weaknesses, by combining their estimations into a unified ML model. This approach is designed to greatly enhance the overall accuracy and robustness of automatic B-point detection.

**Figure 1:**
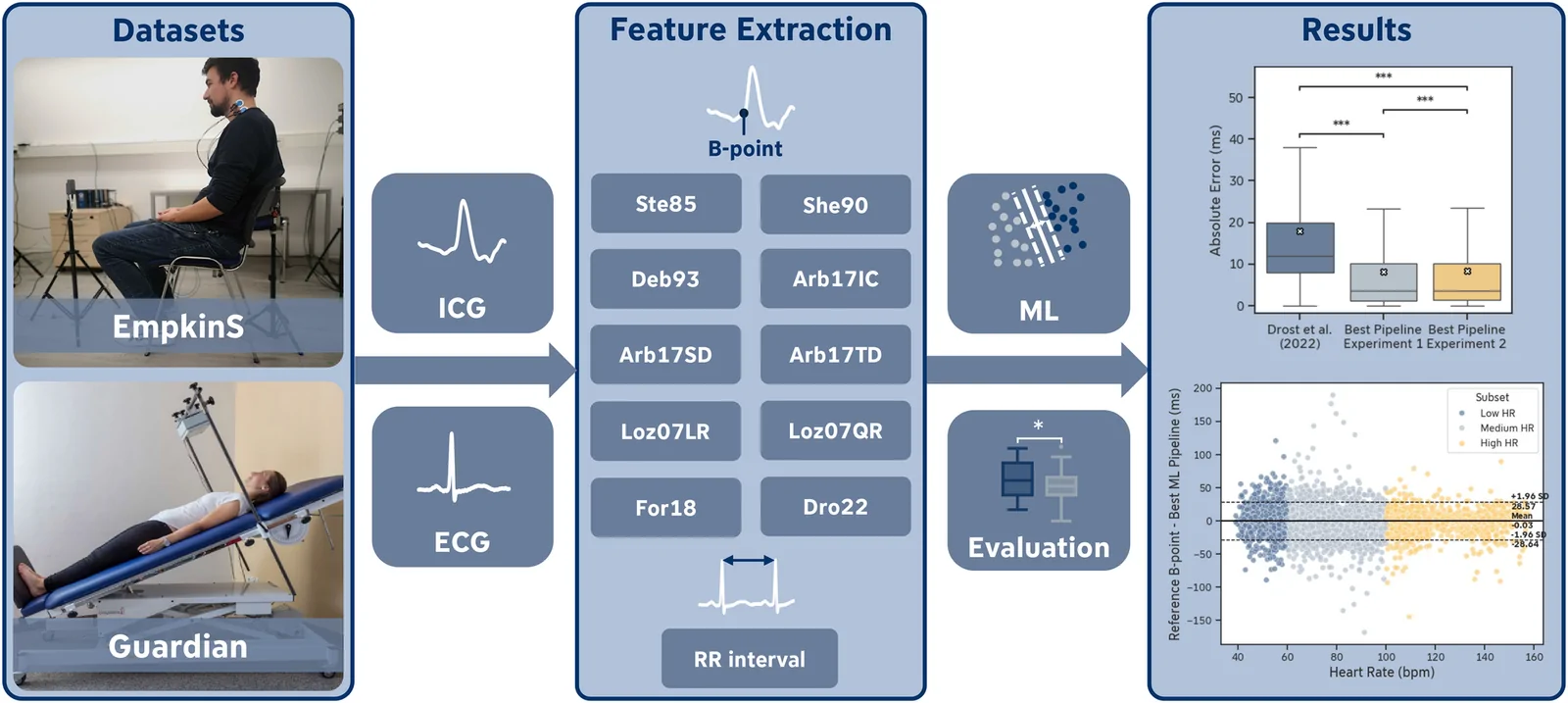
Overview of the main contributions. In this work we present a machine learning-based beat-to-beat detection algorithm of the aortic valve opening from impedance cardiography. Our best performing machine learning model is publicly available in the PEPbench framework, introduced in our previous work.

## 2 Methods

### 2.1 Datasets

The proposed machine learning models were trained and evaluated on data provided by the PEPbench Framework (27). This dataset comprises a total of 11,611 annotated cardiac cycles of synchronized ECG and dZ/dt data from the EmpkinS and Guardian studies. Two independent, trained annotators performed annotations.

The EmpkinS dataset contains 5,000 cardiac cycles, from n=15 healthy participants (60% women) with a mean age of 23.1 ± 2.6 years and a body mass index (BMI) of 21.9 ± 2.4 ${\mathrm{kg}/m}^{2}$ (Mean ± Standard Deviation). Modified versions of the Trier Social Stress Test (TSST), which is a widely used protocol for psychosocial stress induction in the laboratory (32), and a friendly stress-free version of the TSST (f-TSST) (33), were performed on two consecutive days. The standard (f-)TSST protocols involve a 5 min preparation period, followed by two 5 min tasks: a mock job interview and a mental arithmetic task.

The Guardian dataset is a subset of a previously published dataset (34), consisting of 6,611 cardiac cycles from n=24 participants (50% women) with a mean age of 31.2 ± 11.0 years and a BMI of 23.8 ± 3.5 ${\mathrm{kg}/m}^{2}$. Participants were positioned on a tilt table in a supine position, where electrodes for ECG and ICG recordings were attached. The five-phase study began with a 10 min baseline rest. Next, the Valsalva maneuver was performed three times to induce sympathetic activation (35, 36), followed by breathing instructions including breath holding after inhalation, exhalation, and breathing normally. The final two phases constituted an orthostatic challenge: a 10 min 70-degree head-up tilt followed by a 10 min return to the supine position (37, 38).

For both studies, written informed consent was obtained before the study. All procedures adhered to the Declaration of Helsinki and were approved by the local ethics committee of FAU (protocol #493_20 B and protocol #85_15B).

The experimental results from our previous work (27) show physiological states with an average HR of 101.5 ± 24.3 beats per minute (bpm) in the range of 47.2–157.9 bpm, and an average PEP of 88.4 ± 25.0 ms for the EmpkinS dataset, and an average HR of 67.6 ± 13.4 bpm in the range of 39.1–138.3 bpm, and an average PEP of 138.4 ± 27.1 ms for the Guardian dataset. Detailed information on data collection, study protocols, and signal preprocessing is reported in (27).

The two datasets (EmpkinS and Guardian) were assessed separately in previous work. To establish a common baseline for our machine learning experiments, we re-evaluated the performance of the existing methods by combining both datasets. The algorithm proposed by Drost et al. (18) achieved the lowest MAE of 17.9 ± 23.0 ms, followed by the linear regression model of Lozano et al. (1) and the method proposed by Debski et al. (16), with MAEs of 19.6 ± 17.5 ms and 20.5 ± 21.4 ms, respectively. Therefore, we use the method by Drost et al. (18) as a baseline for automatic beat-to-beat B-point detection. Detailed results of other existing methods are provided in the Supplementary Table S1.

### 2.2 Feature extraction

To develop a robust machine learning model for B-point detection, we chose a feature engineering approach that leverages established domain knowledge using our PEPbench framework, which implements 12 published B-point detection algorithms. The estimated B-point locations from each algorithm were combined with the duration of the RR interval to form our feature set. A detailed description of these extracted features is provided in Table 1.

**Table 1:** Overview of features used for training of the machine learning pipelines.

Abbreviation	Citation	Description
Arb17IC	(14)	Last isoelectric crossing of a cardiac cycle before the maximum of the signal in that cardiac cycle (C-Point) (last crossing of the mean of the signal of a cardiac cycle before the C-Point).
Arb17SD	(14)	Peak of the signal in the time window 150 ms to 100 ms before the C-Point.
Arb17TD	(14)	Peak of the signal in a time window starting 300 ms before the C-Point.
Deb93	(16)	Local minimum of the signal with minimal distance to the C-Point.
Dro22	(18)	Point of maximal distance between the signal and a straight line that connects the C-Point and the point of the signal 150 ms before the C-Point.
For18	(7)	Local maximum or zero-crossing in the signal with minimal distance to the C-Point in the most prominent monotonically increasing segment between the A-Point and the C-Point. In case no local maximum or zero crossing can be found, the B-point corresponds to the first point of the segment.
Loz07LR	(1)	Linear regression model RB=0.55⋅RC+4.45 that exploits the close relationship between the RB-Interval and the RC-Interval (R represents the location of the R-Peak in the ECG signal. B and C represent the B-point and C-Point, respectively).
Loz07QR	(1)	Quadratic regression model $\mathrm{RB}=1.233\cdot \mathrm{RC}-0.0032\cdot {\mathrm{RC}}^{2}-31.59$ that exploits the close relationship between the RB-Interval and the RC-Interval (R represents the location of the R-Peak in the ECG signal. B and C represent the B-point and C-Point, respectively).
Mil22	(21)	The segment of the signal prior to the C-point is weighted by a scaling window to enhance the characteristic morphologies of the B-point, which simplifies detection.
Pal21	(8)	B-point is searched in a time window ranging from C-Point—80 ms to the point where half the amplitude of the C-Point is exceeded for the last time before the C-Point. Then the B-point is defined as the closest local minimum or point where a slope of 0.11 is exceeded before the C-Point. If no B-point is found, the slope threshold is decreased, and if B-point detection is still not successful, the minimum of the signal is defined as the B-point.
She90	(3)	Zero-crossing of signal with minimal distance to the C-Point.
Ste85	(17)	Local minimum of signal with minimal distance to the C-Point.
RR interval	(47)	Distance between the R-peak of the current and previous cardiac cycle.

In our previous work, we identified that the B-point detection algorithms within the PEPbench framework were prone to two main types of failure: either a B-point was not detected at all, or its location was physiologically implausible, for example, if a B-point candidate was detected before the R-peak (27), leading to missing values in the dataset. If no B-point was detected by any of the algorithms, we excluded this cardiac cycle. This led to the exclusion of 372 cardiac cycles (3.2%) from the dataset, resulting in a total of 11,239 cardiac cycles. For the remaining cycles, the resulting feature set contained sporadic missing values. Since not all machine learning algorithms can handle missing values, we defined two experiments:

- **Experiment 1** (Missing value inclusion): In this experiment, we restricted the analysis only to ML models that can handle missing feature values (e.g., Random Forest Regressor).
- **Experiment 2** (Missing value imputation): In this experiment, we imputed missing values using the median of valid B-point algorithm outputs within the same cardiac cycle. Cycles with failed RR interval calculation (101 cycles, 0.9%) were excluded to avoid inter-cycle imputation, resulting in a final dataset of 11,138 cardiac cycles. This allows us to additionally include other ML models.

### 2.3 Training and evaluation

Our approach, which combines domain knowledge with an ML pipeline, was evaluated with four different base models: k-Nearest Neighbors Regressor (k-NN), Support Vector Regression (SVR), Decision Tree Regressor (DT), and Random Forest Regressor (RF). To ensure compatibility with distance-based estimators (k-NN and SVR) and those sensitive to feature magnitude, preprocessing integrated two scaling strategies: z-score normalization via StandardScaler and unit-interval rescaling through MinMaxScaler.

Feature selection for Experiment 2 was performed with SelectKBest, which selects the top-K features based on a statistical test (F statistic or mutual information) or SelectFromModel. We did not apply feature selection in Experiment 1, because tree-based regression models (DT and RF) are known to perform feature selection implicitly during their training process (39).

Hyperparameter optimization was performed with grid search for the k-NN and SVR-based pipelines, while randomized search with 4,000 iterations was used for the DT and RF-based pipelines.

To find the best-performing pipeline and the corresponding hyperparameters, we employed a 5-fold nested cross-validation (CV) approach. The inner loop of CV is used to tune the hyperparameters of the pipeline components that are currently evaluated. The outer CV loop is used to evaluate the generalizability of the fine-tuned pipeline combinations. Thereby, the inner CV is exclusively performed on the training set of the outer CV. This design ensures that the final evaluation of the model with the optimized hyperparameters from the inner CV loop is conducted on a completely unseen test set. We enforced a strict participant-level separation: All data belonging to a single participant was kept mutually exclusive between the training and test sets. To assess generalizability beyond the participant-level split used in our primary evaluation, we additionally performed a cross-dataset evaluation: models were trained on one dataset (EmpkinS or Guardian) using the best-performing pipeline configuration from Experiment 1/2, and evaluated on the other, held-out dataset without retraining. This evaluation is particularly relevant given the differing physiological ranges of the two datasets (EmpkinS: PEP range of [143, 499] ms; Guardian: [214, 650] ms), which allows us to probe the extent to which each pipeline can extrapolate beyond the target value range seen during training (about 20% of the EmpkinS samples are outside of the Guardian sample range). Because tree-based regressors are known to be limited in their ability to extrapolate beyond the range of training targets, we additionally evaluated the next best-performing, non-tree-based pipeline from Experiment 2 (MinMaxScaler–SelectKBest–SVR) under the same cross-dataset condition, to determine whether model class affects robustness to distributional shift between datasets.

We evaluated the performance of the ML pipelines using the mean absolute error (MAE) computed against the manually annotated B-point locations and statistically compared the best-performing pipelines obtained in Experiments 1 and 2 to the previously best-performing B-point detection algorithm by Drost et al. (18). To gain more insights into the prediction performance, we conducted further analyses on different subsets of the data using the best-performing model. Given that the Shapiro-Wilk test indicated violations of the assumption of normal distribution across all analyses, we used non-parametric statistical methods on a participant aggregation level.

Firstly, we evaluated potential differences between cardiac cycles where at least one B-point algorithm failed, as we expect B-point detection to be more challenging. Therefore, we divided the dataset into two subsets per experiment and compared the model performance using the Mann-Whitney U test: subset 1 consists of the 10,305 cardiac cycles with no missing values, while subsets 2 contained the remaining 934 (8.3%) and 833 (7.5%) cardiac cycles for Experiment 1 and Experiment 2, respectively.

Secondly, we explored whether model performances depended on the inter-rater variability of the manually labeled ground truth. We categorized the inter-rater agreement into three levels, resulting in three subsets: High agreement is defined as a difference smaller than or equal to 4 ms (66.1% of cardiac cycles), while medium and low agreements correspond to a difference between 4 and 10 ms (17.2%) and larger than 10 ms (16.7%), respectively.

Thirdly, we evaluated the model performances on subsets divided into three heart rate groups (40): Low HR if it is lower than 60 bpm (18.9%), medium HR (between 60 and 100 bpm, 58.8%), and high HR (over 100 bpm, 22.3%).

The statistical differences between inter-rater agreement levels and the grouped HRs (both involving three groups) were assessed using the Kruskal-Wallis H test. Significant results were followed up with post-hoc pairwise comparisons using the non-parametric Wilcoxon signed-rank test. The significance level was set to α=0.05. We accounted for multiple comparisons using the Bonferroni correction. All effect sizes are reported as Hedges’ g. Statistical significance is denoted as: ${}^{*}p<0.05$, ${}^{**}p<0.01$, ${}^{***}p<0.001$.

### 2.4 Availability of data and code

The code used for training and evaluation of the proposed models is publicly available in the GitHub repository of the PEPbench framework (https://github.com/empkins/pepbench) under the MIT license. This framework also provides pipelines for automatic fiducial point detection based on algorithms from related work, along with comprehensive documentation. For further information, we refer to the introductory paper on the framework (27).

The datasets used in this study are publicly available on the Open Science Framework (30, 31). All analyses were performed in Python (v3.12.6), using the package BioPsyKit (v0.13.1) (41), based on pingouin (v0.5.5) (42), and scikit-learn (v1.5.2) (43).

## 3 Results

### 3.1 General model performance

The general model evaluation revealed that the best performance was achieved by a pipeline consisting of a MinMaxScaler, no explicit feature selection, and a RandomForestRegressor. This pipeline achieved an MAE of 8.15 ± 1.14 ms, which was obtained in Experiment 1 (missing values were included). The three best-performing machine learning pipelines per experiment, with their respective mean absolute error (MAE), mean error (ME), and mean absolute relative error (MARE), are listed in Table 2. Detailed results of all pipeline combinations are provided in the Supplementary Table S2.

**Table 2:** B-point detection performance of the three best machine learning pipelines evaluated on the datasets of Experiment 1 and Experiment 2.

		MAE (ms)		ME (ms)	MARE (%)
Subset	Machine learning pipeline	CV-folds	Sample-wise	Sample-wise	Sample-wise
Experiment 1	MinMaxScaler None RandomForestRegressor	8.15 ± 1.14	8.13 ± 12.12	−0.03 ± 14.59	2.46 ± 3.92
	StandardScaler None RandomForestRegressor	8.20 ± 1.14	8.18 ± 12.19	−0.02 ± 14.68	2.47 ± 3.95
	MinMaxScaler None DecisionTreeRegressor	10.08 ± 0.89	10.06 ± 13.72	0.06 ± 17.02	3.02 ± 4.37
Experiment 2	StandardScaler SelectFromModel RandomForestRegressor	8.31 ± 1.06	8.30 ± 12.30	−0.03 ± 14.84	2.51 ± 3.92
	MinMaxScaler SelectKBest RandomForestRegressor	8.35 ± 1.31	8.33 ± 12.30	0.00 ± 14.85	2.53 ± 3.93
	MinMaxScaler SelectFromModel RandomForestRegressor	8.42 ± 1.15	8.40 ± 12.36	−0.06 ± 14.95	2.55 ± 3.97

Mean absolute error (MAE), mean error (ME), and mean absolute relative error (MARE) were evaluated across cross-validation folds and sample-wise. All values are depicted as Mean ± Standard Deviation.

The machine learning pipelines developed in this work consistently and significantly outperformed all traditional B-point detection algorithms, previously evaluated on the same datasets (27). Specifically, RF-based pipelines demonstrated the lowest errors.

In Experiment 1, the pipeline (MinMaxScaler–RandomForestRegressor), which is the overall best-performing pipeline, significantly outperformed the best traditional method by Drost et al. (18) (p<0.001, g=2.23), by reducing the MAE by 54.7% from 17.9 to 8.1 ms. The standard deviation dropped by 47.4% from 23.0 to 12.1 ms.

In Experiment 2, the pipeline (StandardScaler–SelectFromModel–RandomForestRegressor) achieved the lowest MAE of 8.31 ± 1.06 ms. This pipeline is also significantly better than the traditional method proposed by Drost et al. (18) (p<0.001, g=2.17; Figure 2).

**Figure 2:**
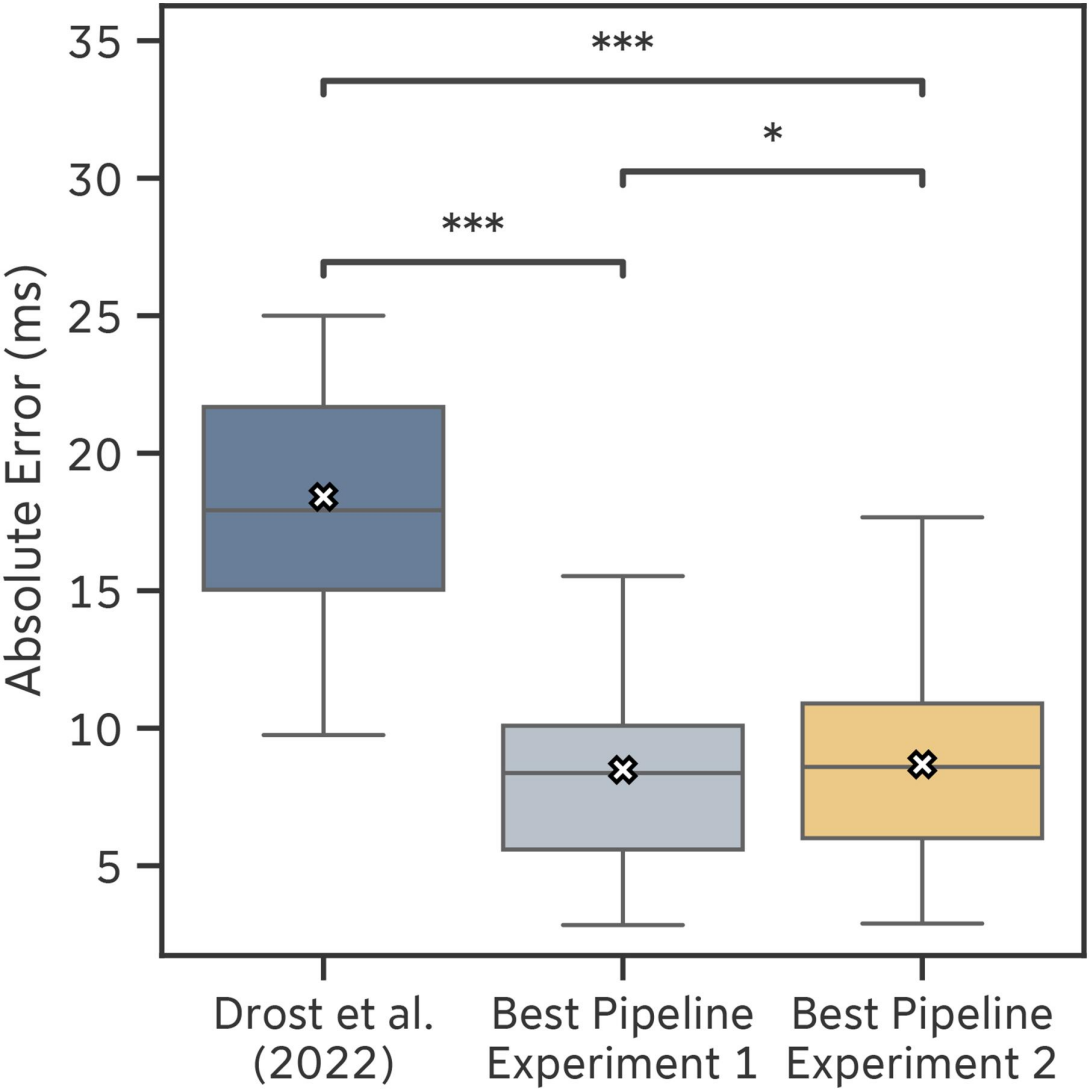
Comparison of the absolute error (ms) of the best-performing classic B-point detection algorithm (18) and the proposed best pipeline for Experiment 1, and Experiment 2. Mean values are denoted by the white cross.

Comparing the best-performing machine learning pipelines of each experiment (missing value inclusion vs. missing value imputation) to each other, a minimal, but significant (p=0.01) difference in MAE of 0.17 ms is obtained. However, the effect size (g=−0.05) is small, indicating high similarity between the outcomes of the two experiments.

Because Experiment 1 included more cardiac cycles and obtained a slightly lower mean absolute error than Experiment 2, the following detailed analyses refer to the best-performing model (MinMaxScaler–RandomForestRegressor) obtained in Experiment 1. The residual plot of this best-performing machine learning pipeline against the manually labeled reference is illustrated in Figure 3.

**Figure 3:**
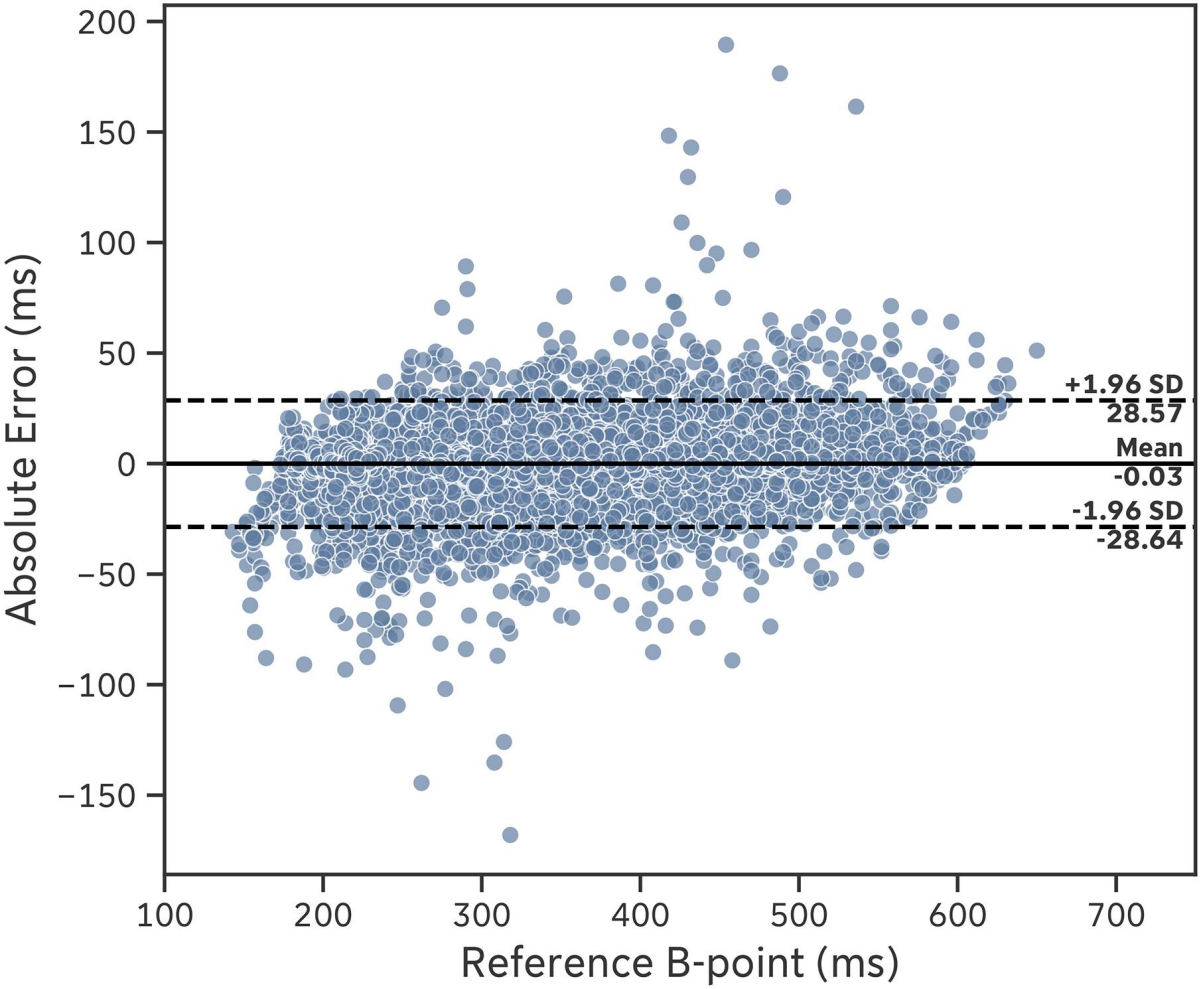
Residual plot of the best performing machine learning pipeline (*MinMaxScaler*–*RandomForestRegressor*).

The error on the subset with a full feature set is slightly but non-significantly lower than the error obtained from the set, where at least one feature detection algorithm failed (7.76 ± 11.42 vs. 12.20 ± 17.60 ms MAE; p=0.41, g=−0.39; Figure 4A).

**Figure 4:**
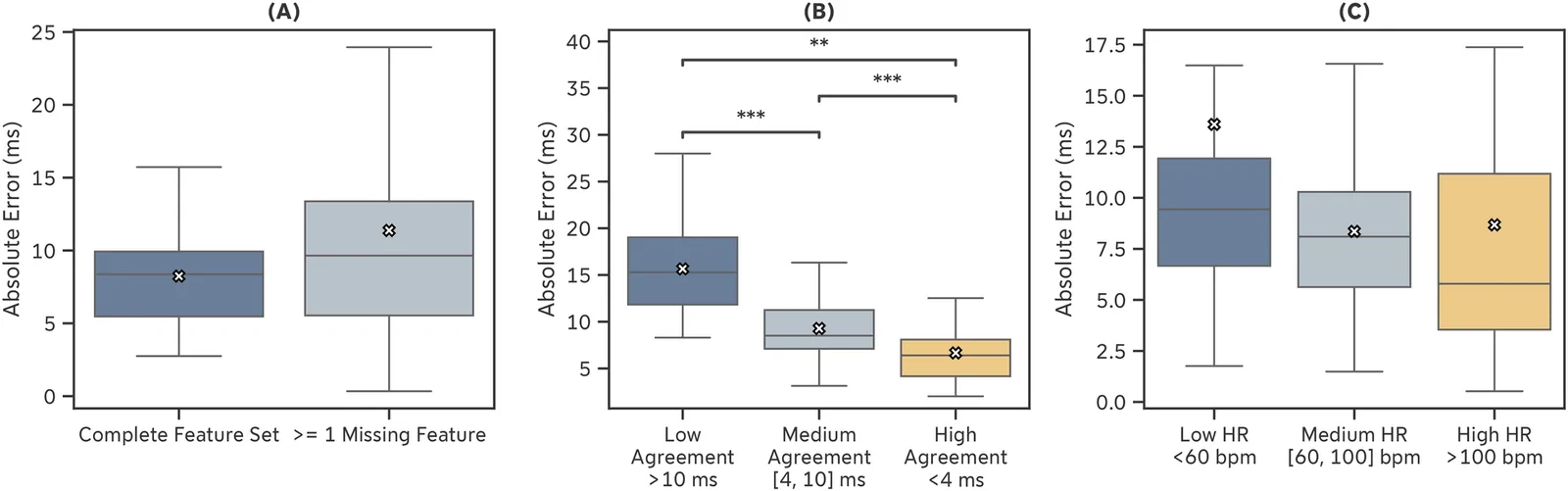
Evaluation of the best performing machine learning pipeline (*MinMaxScaler*-*RandomForestRegressor*) on three subsets of the data. **(A)** Comparison of the subsets where either all features were detected or at least one feature extraction algorithm failed. **(B)** Evaluation on the subsets of low, medium, and high inter-rater agreement. **(C)** Evaluation for different heart rate ranges. Mean values are denoted by the white cross.

Inter-rater agreement influenced model performance, with the error decreasing for higher agreement (low: 15.17 ± 13.76 ms MAE, medium: 9.00 ± 12.76 ms MAE, high: 5.86 ± 9.93 ms MAE) (Figure 4B). The observed changes between all groups are statistically significant (high vs. low: p<0.001, g=−2.10; high vs. medium: p<0.001, g=−0.71; medium vs. low: p<0.001, g=1.50).

The error of the machine learning model across different HRs decreases slightly from low HRs to high HRs (Figure 4C). Statistical analysis revealed no significant differences between the model error of all HR subsets.

Using the annotations of the second annotator as reference values for computing the performance of our proposed model, we obtained a MAE of 10.02 ± 14.54 ms, which is an increased error of 1.67 ms compared to the MAE of 8.35 ± 14.78 ms when evaluated on the labels of the first annotator.

### 3.2 Cross-dataset generalizability

When trained on EmpkinS and evaluated on Guardian, the best-performing Random Forest pipeline (MinMaxScaler–RandomForestRegressor) showed a substantial increase in error (MAE = 36.24 ms) compared to the within-distribution evaluation (MAE = 10.86 ms). The SVR-based pipeline (MinMaxScaler–SelectKBest–SVR), the next best-performing pipeline in Experiment 2, showed comparatively better generalization (MAE = 19.39 ms) under the same cross-dataset condition. Similarly, in the reverse direction (Guardian → EmpkinS), the SVR pipeline (within dataset MAE = 10.33 ms) generalized well, with a subject-wise MAE of 12.70 ± 6.33 ms. The Random Forest pipeline generalized far worse, with a MAE of 52.92 ± 39.35 ms.

### 3.3 Feature importances

Averaging the feature importances of the best-performing model across the cross-validation folds revealed that the model is primarily influenced by the B-point detection algorithm proposed by Drost et al. (18), which had a feature importance score of 53.9. The linear regression model by Lozano et al. (1) was also a critical contributor to the model’s decision-making, with a feature importance of 13.7, followed by the RR interval and the algorithm by Debski et al. (16), with feature importances of 6.8 and 6.1, respectively. The combined feature importance of these four most influential features reached 85.8% of the total feature importance. An overview of all feature importance scores is provided in Table 3.

**Table 3:** SHAP Feature importance scores (Mean ± Standard Deviation) of the best performing machine learning model (MinMaxScaler–RandomForestRegressor) averaged over the 5 cross-validation folds.

Abbreviation	Short description	SHAP importance
Dro22	Point of maximal distance to a straight line between the C-point and the point 150ms before (18)	53.9±2.5
Loz07LR	Linear regression model $R_{B}=0.55\cdot R_{C}+4.45$ (1)	13.7±2.1
RR interval	RR-Interval [derived from (47)]	6.8±1.4
Deb93	Local minimum of second derivative ($d^{2}Z/dt^{2}$) (16)	6.1±0.7
For18	Local maximum or zero crossing in $d^{3}Z/dt^{3}$ signal (7)	4.1±0.6
Ste85	Local minimum of dZ/dt signal with minimal distance to the C-Point (17)	2.6±0.3
Loz07QR	Quadratic regression model $R_{B}=1.233\cdot R_{C}-0.0032\cdot R_{C}^{2}-31.59$ (1)	1.6±0.4
She90	Zero-crossing of dZ/dt signal with minimal distance to the C-Point (3)	1.3±0.2
Arb16TD	Peak of the $d^{3}Z/dt^{3}$ signal in a time window (14)	1.2±0.2
Mil22	Weighted window approach (21)	1.0±0.2
Arb16IC	Last isoelectric crossing of a cardiac cycle before the C-Point (14)	0.7±0.2
Arb16SD	Peak of the $d^{2}Z/dt^{2}$ signal in the time window 150–100 ms before the C-Point (14)	0.6±0.1
Pal21	Rule-based detection in dZ/dt signal (8)	0.2±0.1

The SHAP importance is the mean absolute SHAP value, computed on the held-out test fold.

Further exploring whether the model complexity could be drastically reduced, we retrained the best-performing pipeline using only the top 3 features. The results showed a significant (p<0.001,g=0.17) increase in MAE on CV-fold level (11.73 ± 0.71 ms), compared to the results with the full feature set (8.13 ± 1.14 ms).

## 4 Discussion

In this paper, we introduced and evaluated a novel beat-to-beat B-point detection method using machine learning. Our results show that we can reliably detect the B-point from ECG and dZ/dt signals with a significantly reduced absolute error compared to the best-performing traditional algorithm by Drost et al. (18), from 17.9 ± 23.0 ms to 8.13 ± 12.12 ms.

While the ML models provided in related work (12, 28) achieved lower MAEs than our proposed method, our approach has some key advantages. Most importantly, we performed B-point detection on a beat-to-beat basis. Therefore, we do not rely on ensemble averaging, which substantially simplifies automatic detection by smoothing the dZ/dt signal. Ensemble averaging, and even moving ensemble averaging, may lack sufficient temporal resolution to reflect rapid changes in hemodynamic parameters such as PEP or LVET (6). To our knowledge, only Wang et al. (29) performed ML-based B-point detection on a beat-to-beat basis and achieved an MAE of less than 5 ms with their deep learning model. However, the dataset they used for training and evaluation contained only 417 cardiac cycles, which limits generalizability. Furthermore, the dataset was labeled by a single annotator, and no detailed information on the study population or physiological ranges of the dataset was provided. In contrast, we trained our model on a dataset with more than 11,000 cardiac cycles, labeled by two trained annotators. This allowed us to evaluate our machine learning pipeline on subsets of low, medium, and high inter-rater variability. We also calculated the performance against the annotations of the second rater, which were never seen during training. The achieved MAE of 10.02 ± 14.54 ms indicates that our model can produce results on a comparable level to manual annotation.

To gain a deeper understanding of the best-performing model, further analyses were carried out on different subsets of the data, which represent different B-point detection difficulties. The model exhibited a higher absolute error on the subset where at least one feature extraction algorithm was unable to detect a B-point. Even though the difference showed to be non-significant, a potential explanation could be that the failure of a feature detection algorithm serves as an indicator of an inherently challenging B-point morphology. The inability of an existing algorithm to find a B-point suggests that one or more of its typical defining characteristics might be absent or distorted in that specific cardiac cycle. This leads to a decrease in model performance, explaining the observed increase in absolute error within this subset of data.

Further supporting this hypothesis, inter-rater agreement serves as an additional measure of B-point detection difficulty. As depicted in Figure 4b, the model’s error significantly decreases with increasing inter-rater agreement. This finding indicates that when raters exhibit low agreement on the reference B-point position, the task is inherently more challenging, not only for the human annotators but also for our proposed method. Nevertheless, the performance we obtained for the low agreement subset is on a comparable level to the performance of traditional models for the high agreement subset (15.17 ± 13.76 ms and 16.11 ± 20.67 ms, respectively). Therefore, our approach effectively reduced the error even for difficult-to-detect B-points.

Our results revealed a trend that the absolute error of the B-point detection decreases with higher HRs, as shown in Figure 5. Two potential explanations for this are: Specific B-point morphologies may be more distinct at higher HRs, and the compressed timescale of shorter RR intervals (high HRs) may inherently reduce the potential for a large absolute error. In this scenario, even if the model prediction was incorrect, the predicted B-point location is geometrically closer to the true reference B-point, reducing the potential magnitude of error.

**Figure 5:**
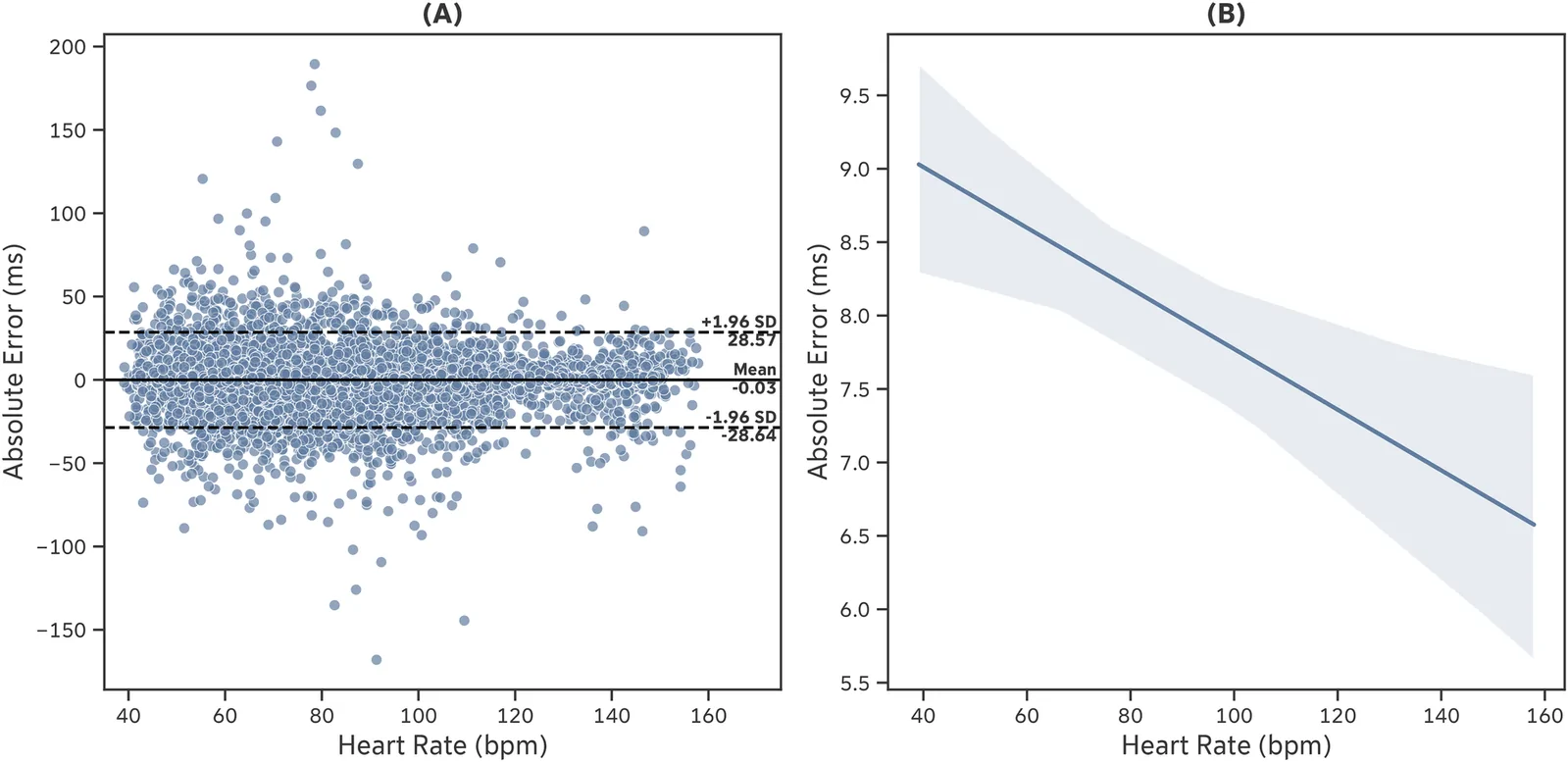
Results from the best performing machine learning pipeline (MinMaxScaler-RandomForestRegressor) for different heart rates. (**A**) Residual plot of the mean absolute error over the heart rate (**B**) Regression plot of the mean absolute error over the heart rate.

In conclusion, even in cases where reliable detection proves more challenging, the proposed machine learning model is still consistently outperforming the algorithm proposed by Drost et al. (18), which showed the best performance in the comparative analysis in our previous work (27). While our analyses revealed that the algorithm proposed by Drost et al. (18) is estimating the actual B-point location too early, which can be seen by the negative MEs of −10.1 ± 18.4 ms (EmpkinS) and −14.8 ± 18.6 ms (Guardian), the proposed machine learning pipeline achieves a ME of −0.03 ± 14.59 ms and therefore reduces this bias to almost zero. Furthermore, the model achieves limits of agreement (±1.96×SD) of −28.64 and 28.57 ms, thereby reducing them compared to the best traditional algorithm (EmpkinS dataset: −46.0 and 29.9 ms) (27).

The error of our proposed model (8.13 ± 12.12 ms) is slightly lower than the inter-rater error (8.35 ± 14.78 ms). Despite the inherent challenges in beat-to-beat B-point detection, this suggests that the performance of our automatic extraction method is on a level comparable to the current standard of manual annotation, while eliminating the bias introduced by individual human raters.

The analysis of feature importance revealed a strong correlation between feature importance and the accuracy of the underlying B-point detection algorithm. While the model is primarily influenced by the algorithms with the lowest error in the benchmarking (27), specifically those proposed by Drost et al. (18), Lozano et al. (1), and Debski et al. (16), algorithms with high errors have less influence on model decision-making. Fusing the algorithms in a single model, even though some of them have minor influence, potentially reduces the large error that some of the algorithms obtain with certain waveforms.

Although there are differences in model performance across different HR ranges, the importance of the RR interval is low. A potential explanation for this is that the RR interval may be a critical indicator only for a small subset of extreme cases. Consequently, while it might provide critical information for these few instances, its overall importance is low when averaged across the entire dataset, where most cardiac cycles fall within a medium RR interval range.

To assess the real-time capabilities of our ML-based approach, we measured the runtime of both its feature extraction and regression steps on an Apple MacBook Pro (M1 Max, 32 GB RAM). Extracting the input features takes 5.1 ms on average. This time is dominated by the slowest single algorithm, Forouzanfar et al. (7), at 2.8 ms. The trained Random Forest regressor adds 6.1 ms per prediction, yielding an end-to-end latency of 11.2 ms per heartbeat. This is well below the average 600–1,000 ms duration of a single heartbeat, confirming the approach is suitable for real-time use. Training the regressor on the full dataset takes 13.5 s, a one-time offline cost, and the resulting model has a serialized size of 90 MB.

The most immediate limitation of our results concerns generalizability, which must be evaluated beyond the current datasets. Given that the datasets mainly comprise data from young and healthy participants, the precision demonstrated is limited to a cohort with minimal hemodynamic complexity. The model’s current validation must be expanded to include groups where physiology is inherently altered. This requires validation on populations from studies with pharmacological interventions (44), and clinical populations, such as major depressive disorder (45), where the B-point is used for PEP computation. As an initial step toward this, we performed a cross-dataset evaluation, training on one dataset and testing on the other. This revealed a substantial increase in error for the Random Forest pipeline (MAE = 36.24 ms), which we attribute to the differing value ranges between datasets (EmpkinS: [143, 499] ms; Guardian: [214, 650] ms) combined with the inability of tree-based models to extrapolate beyond the range of training targets. Notably, the SVR-based pipeline degraded less severely (MAE = 19.39 ms), suggesting that model class choice—not just feature set—matters for out-of-distribution robustness, and that non-tree-based alternatives may be preferable when generalization across differing physiological ranges is a priority.

## 5 Conclusion

In this paper, we introduced a novel machine learning approach for accurate aortic valve opening detection on a beat-to-beat level using the B-point. By building our feature set on existing B-point detection algorithms, we leveraged existing domain knowledge in impedance cardiography analysis to achieve a significant boost in performance over related work. Despite promising advancements in data-driven B-point detection methods (12, 28, 29), limitations remain, such as the reliance on unpublished datasets and the use of ensemble averaging.

In contrast, our proposed algorithm was trained and evaluated on the publicly available PEPbench beat-to-beat datasets and utilizes features that can be easily extracted using the pipelines within that framework (27). This approach provides a straightforward method with results on human rater level, establishing a new benchmark for further developments in this area. Therefore, the accurate and reliable extraction of key time-domain measures, like the Pre-Ejection Period (PEP) and Left Ventricular Ejection Time (LVET), is possible. For practical application, we retrained the best-performing ML pipeline on the full dataset and implemented this model as a new algorithm *BPointExtractionAbelStuehler2026* in the PEPbench framework.

In future work, we plan to evaluate how the performance of the proposed machine learning model generalizes to unseen datasets. Furthermore, deep learning methods for automatic B-point detection could be explored. We hypothesize that these techniques could further improve automated B-point detection, consistent with the notable advancements that have been achieved in other research domains with the application of deep learning.

## Data Availability

The original contributions presented in the study are included in the article/**Supplementary Material**, further inquiries can be directed to the corresponding author.
