# Supplementary material for: Beat-to-beat aortic valve opening detection from impedance cardiography using machine learning
Source: Front Digit Health. 2026 Oct 7;8:1944092. doi: 10.3389/fdgth.2026.1944092 (PMC13685942; doi:10.3389/fdgth.2026.1944092)
Supplement: Supplementary file 1 [file Datasheet1.pdf]

Supplementary Material

1 SUPPLEMENTARY TABLES

Table S1. Evaluation results of the classic B-point detection algorithms against the ground truth labels, combining both EmpkinS and Guardian datasets. All values are depicted as Mean $\pm$ Standard Deviation.

B-point detection algorithm	Abbr.	MAE (ms)	ME (ms)	MARE (%)
Point of maximal distance to a straight line between the C-point and the point 150 ms before (Drost et al., 2022)	Dro22	17.93 ± 23.02	-11.29 ± 26.90	5.34 ± 7.68
Linear regression model $R_B = 0.55R_C + 4.45$ (Lozano et al., 2007)	Loz07LR	19.62 ± 17.46	-10.77 ± 23.96	6.34 ± 6.47
Local minimum of second derivative (Debski et al., 1993)	Deb93	20.47 ± 21.37	-3.03 ± 29.43	5.96 ± 6.47
Local maximum or zero crossing in dZ^3/dt^3 signal (Forouzanfar et al., 2018)	For18	24.40 ± 35.28	18.82 ± 38.54	7.48 ± 11.31
Weighted window approach (Miljković & Šekara, 2022)	Mil22	24.93 ± 35.19	7.32 ± 42.51	7.48 ± 11.65
Quadratic regression model $R_B = 1.233R_C - 0.0032R_C^2 - 31.59$ (Lozano et al., 2007)	Loz07QR	25.77 ± 25.88	4.96 ± 36.18	7.72 ± 8.16
Zero-crossing of dZ/dt signal with minimal distance to the C-Point. (Sherwood et al., 1990)	She90	19.19 ± 20.05	-2.49 ± 35.33	8.41 ± 5.67
Last isoelectric crossing of a cardiac cycle before the C-Point (Arbol et al., 2016)	Arb16IC	30.79 ± 26.47	0.04 ± 40.60	9.05 ± 8.14
Local minimum of dZ/dt signal with minimal distance to the C-Point (Stern et al., 1985)	Ste85	30.93 ± 41.69	28.59 ± 43.33	9.67 ± 13.76
Peak of the dZ^2/dt^2 signal in the time window 150ms to 100ms before the C-Point. (Arbol et al., 2016)	Arb16SD	44.63 ± 28.70	40.63 ± 34.12	13.54 ± 10.11
Peak of the dZ^3/dt^3 signal in a time window (Arbol et al., 2016)	Arb16TD	44.78 ± 55.22	11.24 ± 70.21	13.30 ± 18.71
Rule-based detection in dZ/dt signal (Pale et al., 2021)	Pal21	105.45 ± 113.31	71.90 ± 137.08	29.41 ± 29.32

Table S2. B-point detection performance of all machine learning pipelines evaluated on the datasets of Experiment 1 (Missing values included) and Experiment 2 (Median Imputation). Mean absolute error (MAE), mean error (ME), and mean absolute relative error (MARE) were evaluated across cross-validation folds and sample-wise. All values are depicted as Mean $\pm$ Standard Deviation.

		MAE (ms)		ME (ms)	MARE (%)
**Experiment**	**Machine Learning Pipeline**	**CV-folds**	**Sample-wise**	**Sample-wise**	**Sample-wise**
**Experiment 1**	MinMaxScaler None RandomForestRegressor	8.15 ± 1.14	8.13 ± 12.12	-0.03 ± 14.59	2.46 ± 3.92
	StandardScaler None RandomForestRegressor	8.20 ± 1.14	8.18 ± 12.19	-0.02 ± 14.68	2.47 ± 3.95
	MinMaxScaler None DecisionTreeRegressor	10.08 ± 0.89	10.06 ± 13.72	0.06 ± 17.02	3.02 ± 4.37
	StandardScaler None DecisionTreeRegressor	10.20 ± 1.01	10.18 ± 14.35	-0.13 ± 17.59	3.05 ± 4.67
**Experiment 2**	StandardScaler SelectFromModel RandomForestRegressor	8.31 ± 1.06	8.30 ± 12.30	-0.03 ± 14.84	2.51 ± 3.92
	MinMaxScaler KBest RandomForestRegressor	8.35 ± 1.31	8.33 ± 12.30	0.00 ± 14.85	2.53 ± 3.93
	MinMaxScaler SelectFromModel RandomForestRegressor	8.42 ± 1.15	8.40 ± 12.36	-0.06 ± 14.95	2.55 ± 3.97
	StandardScaler SelectFromModel RandomForestRegressor	8.42 ± 1.15	8.40 ± 12.36	-0.06 ± 14.95	2.55 ± 3.97
	StandardScaler SelectKBest RandomForestRegressor	8.43 ± 1.07	8.42 ± 12.60	-0.06 ± 15.15	2.55 ± 3.99
	StandardScaler SelectKBest SupportVectorRegressor	8.48 ± 1.26	8.47 ± 15.93	-0.20 ± 18.04	2.59 ± 5.42
	MinMaxScaler SelectFromModel DecisionTreeRegressor	8.64 ± 1.31	8.63 ± 14.69	-0.09 ± 17.04	2.63 ± 4.60

MinMaxScaler SelectKBest DecisionTreeRegressor	8.81 ± 1.21	8.79 ± 15.78	0.08 ± 18.06	2.67 ± 4.79
StandardScaler SelectFromModel DecisionTreeRegressor	8.80 ± 1.21	8.79 ± 14.67	-0.27 ± 17.10	2.69 ± 4.64
StandardScaler SelectFromModel SupportVectorRegressor	8.81 ± 1.11	8.80 ± 13.30	-0.15 ± 15.95	2.64 ± 4.10
StandardScaler SelectKBest DecisionTreeRegressor	9.01 ± 1.30	9.00 ± 15.52	0.20 ± 17.94	2.71 ± 4.70
MinMaxScaler SelectKBest KNeighborsRegressor	9.40 ± 1.00	9.39 ± 14.91	0.08 ± 17.62	2.86 ± 4.98
StandardScaler SelectKBest KNeighborsRegressor	9.43 ± 1.00	9.42 ± 14.84	0.17 ± 17.57	2.87 ± 4.95
MinMaxScaler SelectFromModel SupportVectorRegressor	9.47 ± 1.28	9.45 ± 14.47	-0.19 ± 17.29	2.83 ± 4.44
MinMaxScaler SelectKBest SupportVectorRegressor	9.47 ± 1.29	9.46 ± 14.60	-0.42 ± 17.39	2.84 ± 4.57
StandardScaler SelectFromModel KNeighborsRegressor	9.54 ± 0.94	9.53 ± 13.16	0.13 ± 16.25	2.85 ± 4.16
MinMaxScaler SelectFromModel KNeighborsRegressor	9.64 ± 0.90	9.63 ± 13.04	0.08 ± 16.21	2.87 ± 4.14
